# Osteopetrosis and Its Relevance for the Discovery of New Functions Associated with the Skeleton

**DOI:** 10.1155/2015/372156

**Published:** 2015-03-19

**Authors:** Amélie E. Coudert, Marie-Christine de Vernejoul, Maurizio Muraca, Andrea Del Fattore

**Affiliations:** ^1^Institut National de la Santé et de la Recherche Médicale U1138, Centre de Recherche des Cordeliers, Paris, France; ^2^Institut National de la Santé et de la Recherche Médicale U1132, Hôpital Lariboisière, Paris, France; ^3^Regenerative Medicine Unit, Bambino Gesù Children's Hospital, IRCCS, Piazza Sant'Onofrio 4, 00165 Rome, Italy

## Abstract

Osteopetrosis is a rare genetic disorder characterized by an increase of bone mass due to defective osteoclast function. Patients typically displayed spontaneous fractures, anemia, and in the most severe forms hepatosplenomegaly and compression of cranial facial nerves leading to deafness and blindness. Osteopetrosis comprises a heterogeneous group of diseases as several forms are known with different models of inheritance and severity from asymptomatic to lethal. This review summarizes the genetic and clinical features of osteopetrosis, emphasizing how recent studies of this disease have contributed to understanding the central role of the skeleton in the whole body physiology. In particular, the interplay of bone with the stomach, insulin metabolism, male fertility, the immune system, bone marrow, and fat is described.

## 1. Introduction 

Bone is a dynamic tissue which undergoes continuous self-renewal, and bone homeostasis relies on functional equilibrium among three types of cells: osteoclasts essential for bone resorption, osteoblasts responsible for bone matrix formation, and osteocytes involved in the reception and transduction of mechanical stimuli and in the regulation of osteoclast/osteoblast differentiation and function [1]. The balance between bone synthesis and resorption is finely tuned and any perturbations of this balance in adults trigger bone disease. Human osteopetrosis was first described by Albers-Schönberg in 1904 [2]. Osteopetrosis (*osteo*: bone and* petros*: stone) regroups a set of rare, heterogeneous, and inherited bone diseases characterized by increased bone mass. Osteopetrosis is therefore an osteocondensing disease. In principle, two causes could give rise to this osteocondensing phenotype: increased bone formation or failure of resorption by osteoclasts. However, osteopetrosis is known to result from defective osteoclast differentiation or function [3, 4].

Important progress has been made during the past decades in understanding the molecular mechanisms underlying the development of hereditary diseases characterized by increased bone mass [3, 5].

Our objective in this review is not to give a detailed description of all the sclerosing bone diseases; such information can be found in other reviews [3, 4, 6, 7]. Instead, we discuss recent findings regarding osteopetrosis and how the study of this disease has contributed to new understanding of functions associated with the skeleton [8–10].

## 2. Osteoclasts

Osteoclasts are highly specialized cells responsible for the dissolution of bone mineral and for the degradation of organic matrix. This activity is essential to bone remodeling and mineral homeostasis [8].

Osteoclasts are multinucleated cells (containing up to 50 nuclei), derived from the fusion of mononuclear cells belonging to the monocyte-macrophage lineage. Under the influence of factors secreted by osteoblasts and/or stromal cells present in the bone microenvironment, these precursors differentiate into osteoclasts [1].

The osteoclast differentiation pathway and the molecules involved are now well established. M-CSF (macrophage colony stimulating factor) is expressed by osteoblasts and binds the c-fms receptor on osteoclast precursors, stimulating their proliferation and the expression of RANK (receptor activator of NF-*κ*B) receptor. The interaction of RANK-L, expressed and secreted by osteoblasts and stromal cells, with its receptor propels the fusion of osteoclast progenitors to form a giant multinucleated cell. Osteoprotegerin inhibits osteoclast differentiation by acting as a receptor decoy for RANK-L [1].

A terminally differentiated osteoclast is able to degrade extracellular bone matrix by the action of specific proteins. To resorb bone matrix, osteoclasts must be perfectly polarized with a ruffle border and a sealing zone. These two features allow the creation of a resorption lacuna into which H^+^ ions are actively secreted in order to acidify it, leading to dissolution of bone matrix hydroxyapatite [1]. Creation of the acidic compartment requires a continuous source of protons. Type II carbonic anhydrase (CAII) hydrates CO_2_ to form carbonic acid, which spontaneously dissociates into protons and HCO_3_^−^ ions. The protons are actively transported into the resorption lacuna by a vacuolar ATPase proton pump located in the ruffled border domain [1, 11]. The HCO_3_^−^ ion is exchanged with Cl^−^ by a bicarbonate/chloride antiport on the basolateral membrane of the cell. The chloride ion is translocated into the resorption lacuna through chloride channel 7 (ClCn7), recently reclassified as chloride/proton antiport. The acidic environment promotes the dissolution of inorganic content and also exposes the organic matrix, which is then ready to be digested by secreted proteolytic enzymes [1, 11].

The collagenous bone matrix is dissolved by two groups of enzymes, the matrix metalloproteases and the lysosomal cathepsins. Cathepsin K especially has been identified as a key enzyme in osteoclast function. This enzyme is secreted into the resorption lacuna and degrades type I collagen in the acidic environment [12].

The acquisition and maintenance of osteoclast membrane polarity require a complex system of vesicle trafficking and ongoing cytoskeletal renewal [1]. One of the proteins involved in these processes is Plekhm1 (pleckstrin homolog domain containing family M with run domain member 1). This protein plays a crucial role in acidification and trafficking of intracellular vesicles [13, 14]. A recently discovered protein important for osteoclast trafficking activity is Snx10 (sorting nexin 10). Snx10 belongs to a family of about 30 proteins sharing the PX (phox homology) phospholipid binding domain and is involved in protein trafficking and osteoclast differentiation/function [15, 16].

## 3. Osteopetrosis

Osteopetrosis is a generic name for a group of rare genetic bone diseases characterized by osteoclast failure [6]. Several forms are known with different models of inheritance and severity. The adult autosomal dominant type II form or Albert-Schönberg disease classified as mild is sometimes associated with bone symptoms. This is the most frequent form of osteopetrosis observed by rheumatologists. In contrast, the infantile recessive osteopetroses are severe forms and usually lethal in childhood without treatment [3, 5–7, 17]. Mutations in at least 8 genes (Table 1) have been identified as being responsible for osteopetrosis pathogenesis in humans.

### 3.1. Autosomal Recessive Osteopetrosis

Autosomal recessive osteopetrosis is a severe disease diagnosed in the first months of life owing to a variety of problems [3]. Patients are treated in pediatrics or hematology departments. Sick children have recurrent infections. They also show bruising and frequent bleeding secondary to medullar hyperplasia caused by bony invasion of the medullar space. Cranial nerve compressions can occur leading to blindness and deafness. Neurological defects may also be observed in some patients independently of nerve compressions. X-ray analysis reveals dense bones which are characterized by extreme brittleness. Untreated children usually die during their decade from hemorrhage, pneumonia, anemia, or infection. Hematopoietic stem cell transplantation (HSCT) is the only treatment option currently far available [3, 5, 18].

**Figure 1:**
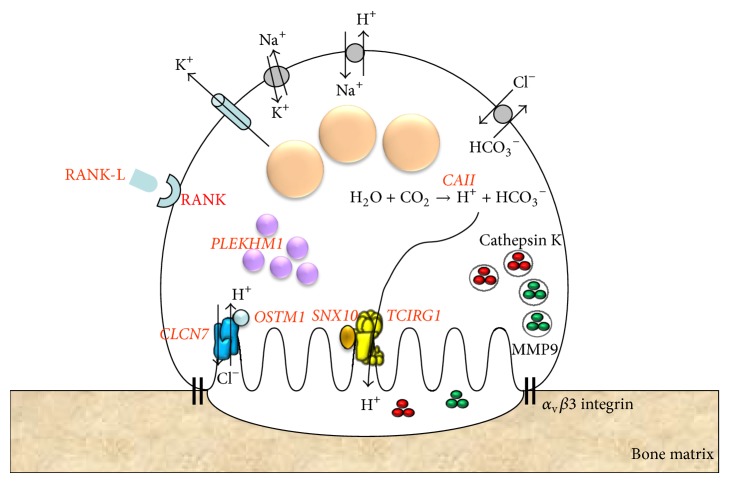
Schematic diagram showing an osteoclast and the involved genes in osteopetrosis. Cellular localization and protein involved in osteoclast differentiation and function. The genes mutated in human osteopetrosis are red in bold.

Several biological abnormalities can cause this pathology. Generally in ARO, the number of osteoclasts is normal or high, but their acidifying activity, compulsory for bone resorption, is impaired [17]. Several genes are known to be involved in this form of osteopetrosis (Table 1, Figure 1). About 50% of ARO patients harbors loss-of-function mutations of* TCIRG1* which codes for the proton pump V-ATPase *α*3 subunit [17, 19]. Loss-of-function mutations of the* CLCN7* gene, coding for chloride channel 7, have also been described in ~10% of ARO patients [20]. Mutations in* OSTM1* (osteopetrosis associated transmembrane protein 1), coding for a protein involved in transport of ClCn7 to the ruffled border (and considered as a *β* subunit of ClCn7), have been described as causing severe osteopetrosis in ~5% of patients [21–23]. Primary neurological defects can also be present in patients bearing* OSTM1* or* CLCN7* mutations [20, 23].

Two cases of intermediate forms of ARO caused by* PLEKHM1* mutations have been described. An “Erlenmeyer flask” deformity of the distal femora, bone pain, and chondrolysis of the left hip were described in one patient. Interestingly, a brother with the same mutation showed no clinical signs [14]. Recently, a mutation in the* SNX10* gene was found in 15 families in which the patients displayed a heterogeneous phenotype. Mild growth retardation, hypocalcemia, hydrocephalus, severe hematological abnormalities, and visual impairment have been described in patients with loss of function mutations of* SNX10* [3, 15, 16, 24].

Less than 4% of ARO patients harbors loss-of-function mutations of* TNFSF11*, encoding RANK-L, or of* TNFRSF11A*, encoding RANK receptor, and constitute a distinct subgroup of recessive osteopetrosis. Indeed, bone biopsies from these patients revealed a complete lack of osteoclasts [25–27]. In addition, patients with* TNFSF11* mutations exhibit some immune abnormalities and not palpable lymph nodes, but B and T lymphocyte numbers are normal. By contrast, most of the patients with* TNFRSF11A* mutations have a more severe immunological phenotype with a defect in memory B lymphocyte differentiation and a reduction in immunoglobulins levels [25–27].

Treatment of most recessive forms of osteopetrosis includes HSCT, which restores osteoclast function. However, osteopetrosis caused by* TNFSF11* mutations cannot be treated by HSCT, because an osteoblast defect is the basis of this pathology [28]. In practice, a molecular genetic diagnosis should be made before transplantation to ensure that the pathology is not due to a RANK-L mutation.

### 3.2. A Specific Intermediate Recessive Osteopetrosis (IRO): Type II Carbonic Anhydrase Deficiency

In 1983, an autosomal recessive osteopetrosis syndrome associated with renal tubular acidosis was described [29]. The clinical signs of the affected patients are highly variable. Mental deficiency is frequent, but not always present. Optical nerve compression and dental malocclusions can occur. Renal tubular acidosis can explain the hypotonia, apathy, and muscular weakness occurrence in some patients. By radiography CAII deficiency resembles other forms of osteopetrosis, but brain calcifications can develop during childhood and osteosclerosis and bone modeling spontaneously decrease instead of increasing in the course of pathology evolution. Metabolic acidosis occurs during the neonatal period, and renal tubular acidosis, both proximal and distal, has been described [29, 30].

CAII is expressed in many different tissues including brain, kidney, red blood cells, cartilage, lung, and digestive mucosa. All patients with this pathology have a selective defect involving CAII expressed in erythrocytes [31].

### 3.3. Type II Autosomal Dominant Osteopetrosis (ADOII Also Known as Albert-Schönberg Disease)

ADO II is commonly called benign osteopetrosis but presents with an extremely heterogeneous course from asymptomatic to rarely fatal. Prevalence of the pathology has been estimated at 5 per 100 000 [32].

ADOII clinical and radiological signs occur quite late in childhood or in the teens, although earlier occurring has sometimes been reported. ADOII patients usually displayed osteosclerosis at the vertebral level (so-called sandwich vertebrae) and also a bone in bone aspect observed mainly in the iliac bones, but sometimes in other epiphyses. An increase in cranial bone density can also occur. In addition, on radiography, alternating dense and light bands are often seen in iliac bones and at the extremities of long bones [7, 33].

The main ADOII complications involve the skeleton [34]. Bone fractures occur in 80% of patients, with a mean of 3 fractures per patient. A few patients have had more than 10 fractures. The femur is the most fractured bone in this pathology, but fractures can occur on any long bones and even at the posterior arch of the vertebrae, which often leads to a spondylolisthesis. Scoliosis is not rare. Hip arthritis is frequent (in 50% of the cases) and could be due to excessive stiffness of the subchondral bone. Arthritis can occur in other locations as well. Mandibular osteomyelitis is often associated with dental abscess or carious cavity. Cranial nerve compressions caused by osteosclerosis are rare. Auditory or visual impairment occurs in less than 5% of affected individuals [7, 33].

Orthopedic treatment is often necessary to treat fractures and arthritis. Arthropathies are technically difficult and postsurgical complications, such as strengthening delay, infections, and pseudoarthritis are frequent (50% of cases) due to bone stiffness. The penetrance of ADOII is 60–90%. Disease severity is highly variable, even within the same family [33]. For example, in 3 families in which most of the affected individuals expressed only a mild form of ADOII, some members exhibited anemia and blindness caused by optical nerve compression. This phenotype has been called intermediate osteopetrosis because of its overlap with that of mild ARO [33].

About 70% of patients affected by ADOII harbors heterozygous dominant negative mutations of the* CLCN7* gene (Figure 1, Table 1) [33]. In the remaining ~30% of cases, no mutations in* CLCN7* gene sequences were found, suggesting involvement of further genes in the pathogenesis of this form of osteopetrosis [33].

## 4. The Relevance of Osteopetrosis Studies to New Understanding of Functions Associated with the Skeleton

### 4.1. Osteopetrosis and the Bone-Stomach Interaction

Osteopetrorickets is a bone disorder characterized by increase of bone mass with a defect of skeletal mineralization. Schinke and coauthors performed histological analysis of undecalcified bone biopsies of 21 patients who received a diagnosis of osteopetrosis. In patients with loss-of-function mutations in the* TCIRG1* gene, an increase of unmineralized bone matrix osteoid was observed. The same pathological enrichment of osteoid was confirmed in* oc/oc* mice carrying a loss-of-function mutation of the* tcirg1* gene, while no increase was revealed in osteopetrotic* scr* ^−/−^ mice [35].

The increase of osteoid volume was associated with hypocalcemia, due to a defect of intestinal calcium uptake. Indeed it was shown that* TCIRG1* is also expressed in the fundus, a region of the stomach involved in gastric acidification, and loss-of-function mutations induce hypochlorhydria and reduced intestinal calcium uptake in both humans and mice [35].

This study was fundamental in demonstrating a physiological link between the stomach and bone. Gastric acidification is a prerequisite for efficient intestinal calcium uptake; in hypochlorhydria, intestinal calcium uptake is lowered leading to parathyroin hormone (PTH)-dependent activation of osteoclasts and an osteoporosis phenotype. In the case of loss-of-function mutation of* TCIRG1*, intestinal calcium uptake is reduced and PTH-dependent stimulation of bone resorption is blocked, resulting in an osteopetrorickets phenotype [35].

Barvencik et al. performed histomorphometric analysis of bone biopsies of 9 osteopetrotic patients with loss-of-mutation in the* TCIRG1*,* CLCN7,* and* TNFSF11A* genes [36]. Pathological enrichment of nonmineralized bone matrix was observed in all cases with* TCIRG1* mutations. In contrast, there was no sign of osteopetrorickets in patients with* CLCN7 *and* TNFSF11A* gene mutations [35–37].

### 4.2. Osteopetrosis and Insulin Metabolism

Osteopetrosis studies were fundamental to understand the link between bone and osteocalcin signaling. Osteocalcin is a small protein embedded in bone matrix. Osteocalcin can exist in two different forms, undercarboxylated and carboxylated on 3 glutamic acid residues [10, 38]. The carboxylated form has high affinity for the hydroxyapatite, facilitating its engraftment in the bone matrix. It was shown that acidic pH can decarboxylate proteins [39]. Ferron et al. investigated whether acidic bone resorption lacuna promotes the decarboxylation of osteocalcin. Indeed they observed that in* oc/oc* mice the levels of undercarboxylated osteocalcin were reduced by 30% compared to wild-type animals. Similar features were observed in wild-type mice that received fetal liver hematopoietic stem cells (HSCs) from* oc/oc* mice confirming the relevance of osteoclast function in osteocalcin-insulin signaling. Moreover they observed that* oc/oc* mice were glucose intolerant, with reduced serum insulin levels, pancreas insulin content, and insulinexpression in the pancreas [40, 41].

Interestingly, it was shown that osteopetrotic patients affected by autosomal dominant osteopetrosis with osteoclast acidification defects have lower levels of insulin and a lower undercarboxylated/carboxylated osteocalcin ratio but diabetes was not reported [40, 41].

### 4.3. Osteopetrosis and Male Fertility

Osteocalcin is very important for the cross talk between bone and the systems responsible for male fertility [42, 43]. Karsenty's group showed that osteocalcin is able to stimulate, in a cAMP response element binding (CREB) protein-dependent manner, the production of testosterone by testes. This function is mediated by the interaction of osteocalcin with GPRC6A, a G-coupled receptor expressed in Leydig cells [42, 43].

In 1997 Cohen et al. [44] showed that op/op mice (which lack colony stimulating factor 1, CSF-1) have reduced mating ability, low sperm numbers, and low serum testosterone levels due to decreased Leydig cell steroidogenesis. The study also showed how CSF-1 is essential for the development and function of the hypothalamic-pituitary-gonadal axis. Further studies in osteopetrotic animal models will be important to confirm the interaction between bone and male fertility.

### 4.4. Osteopetrosis and the Immune System

It now well established that there is a tight correlation between bone and the immune system, which has led to a new discipline called osteoimmunology. This research area is just now expanding and we are beginning to better understand the relevance of this interplay in bone diseases [45].

Many osteopetrotic animals are characterized by immunological defects. Associated defects in B cell function were attributed to mutations in genes involved in osteoclast differentiation or function or to an abnormal medullary microenvironment.* oc/oc* mice display a block at the pro-B to pre-B cell transition, which is due to a defect of the bone microenvironment rather than to a cell autonomous defect of B cells, because* in vitro* experiments showed that B cell progenitors isolated from osteopetrotic mice were able to differentiate into immature B cells [46].

Moreover it was shown that *rankl* ^−*/*−^ mice display an immunological defects. Apart the alterations of B cell differentiation, Kong and coauthors described a reduction in thymus size and a block of thymocyte development at the CD4^−^CD8^−^CD44^−^CD25^+^ stage [47]. These effects are correlated with many functions exerted by RANKL in the immune system [48].

Many studies have been published regarding the effects on osteoclast differentiation and function following alterations of immune cells [45]. The involvement of T regulatory cells (Treg) is still under investigation. In particular, it was shown that animal overexpressing the transcription factor FoxP3 (forkhead box P3) displayed osteopetrotic phenotype with increased bone mass and reduced osteoclast number and activity [49]. Moreover* in vitro* experiments suggested that Treg cells could inhibit osteoclast differentiation and function by suppression of cytoskeletal reorganization [50].

### 4.5. Osteopetrosis and Bone Marrow

Bone and bone marrow can be considered as two distinct compartments of the same functional unit, the bone-bone marrow organ. Perturbations to one of the compartments typically affect the other as well [51].

Indeed it was shown that dysfunction of osteoclast activity results in aberrant formation of the HSC niche, leading to retention of HSC in the spleen. The frequency and absolute number of LinnegSca1^+^cKit^+^ (LSK cells) were decreased by 90% and 99.8%, respectively, in the bone marrow of* oc/oc *mice compared to controls. This alteration was associated with a defect of mesenchymal stem cells to differentiate into osteoblasts. The effect was revealed by a dramatic reduction in the expression of the osteoblast markers* Runx2*,* Alp*,* Osteocalcin* and* Bsp *and a reduced proportion of cells expressing CD51 and the integrin *α*5 (CD49e). The study showed that osteoclasts promote the formation of the HSC niche, regulating the osteoblast differentiation important for the niche [52]. Indeed the authors showed that the absence of osteoclast activity affects formation of the bone marrow HSC niche and impairs ability of mesenchymal stem/stromal cells to recruit hematopoietic progenitor cells. Moreover the restoration of osteoclast function by treatment with CD45^+^Sca1^+^ cells reestablishes normal levels of hematopoietic progenitors in the bone marrow [46, 53].

### 4.6. Osteopetrosis and Fat

The relationship between bone and adipose tissue is an area of intensive investigations because molecules involved in bone-fat interactions could be used as pharmacological targets to prevent osteoporosis and bone fractures [54]. In particular, the involvement of peroxisome proliferator-activated receptor-*γ* (PPAR-*γ*) was studied. PPAR-*γ* is a nuclear receptor and acts as a heterodimer with retinoid X receptor. Ligands for PPAR-*γ* include long-chain fatty acid and synthetic compounds such as thiazolidinedione [55]. PPAR-*γ* functions are associated with activation of the adipogenesis and inhibition of the osteoblastogenesis [56, 57].

Moreover Wan et al. investigated PPAR-*γ* function in osteoclasts [58]. The authors used TieCre/flox mice to delete PPAR-*γ* in osteoclasts. These mice developed increased bone mass with a parallel reduction of bone marrow cavities and extramedullary hematopoiesis. Indeed deletion of PPAR-*γ* resulted in impaired osteoclast differentiation and activity, since it regulates c-fos expression involved in RANKL signaling [58].

Moreover Cock et al. demonstrated that the absence of PPAR-*γ* in white adipose tissue led to lipodystrophy, increased bone mineral density, and extramedullary hematopoiesis in spleen [59]. This interplay between bone and adipose tissue has clinical important implications, since a long-term treatment with the PPAR-*γ* agonist rosiglitazone in patients affected by type 2 diabetes could result in osteoporosis and bone fractures [54, 58].

## 5. Conclusion

Rare hereditary diseases inducing a bone condensation have shed new light on several aspects of bone cellular biology that were not well known. Indeed the study of these diseases allowed the identification of new mechanisms of osteoclast differentiation and function and the discovery of new functions associated with the skeleton. Much evidence suggests that the skeleton has a central role in bone physiology since bone disorders usually impact other organs [8, 9, 42, 43, 45, 53, 54, 60]. Osteopetrosis studies were essential to demonstrate these interactions. However, there are some features of these diseases that require further investigation. For example, as in other monogenic diseases, the genotype-phenotype correlation is not always clear and consistent. Indeed, the same mutations can give rise to different phenotypes, as exemplified by the* CLCN7* gene heterozygous mutations. Moreover, the mutations identified to date explain only 70% of osteopetrosis cases. Efforts to identify the mutations responsible for the remaining 30% are on-going [33].

From a pathophysiological point of view, it is worth noting that the pathologies caused by reduced osteoclastic activity such as osteopetrosis lead to frequent fractures. This might be linked to a skeleton elasticity defect, but also to an inability to repair micro damage in bones because of a lower rate of bone turnover. This situation illustrates the well-known discrepancy between the bone quantity and its resistance to mechanical stress. In contrast, pathologies caused by an increase in bone formation due to increased activity of the Wnt signaling pathway (striated osteopathy) or to TGF*β* activating mutations (Camurati Engelmann disease) are not associated with an increased incidence of fractures [7].

In conclusion, further study of osteopetrosis will allow us to better understand the physiology of bone and its impact on the whole body. Moreover our challenge for the future will be to identify new therapeutic approaches for this disabling disease, particularly for those forms for which only palliative intervention is currently available.

## Figures and Tables

**Table 1:** Genes mutated in osteopetrotic patients.

Osteopetrosis form	Genetic transmission	Gene	Mutation type	Protein
ARO	Autosomal recessive	*TCIRG1 *	Loss of function	*α*3 subunit V-ATPase
		*CLCN7 *	Loss of function	Chloride channel 7
		*OSTM1 *	Loss of function	Osteopetrosis associated transmembrane protein
		*PLEKHM1 *	Loss of function	Pleckstrin homology domain containing family M, member I
		*SNX10 *	Loss of function	Sorting nexin 10
		*TNFSF11 *	Loss of function	Receptor activator for nuclear factor *κ*B ligand
		*TNFRSF11A *	Loss of function	Receptor activator for nuclear factor *κ*B

IRO	Autosomal recessive	*CAII *	Loss of function	Carbonic anhydrase II

ADO II	Autosomal dominant	*CLCN7 *	Dominant negative	Chloride channel 7
